# Scaphoid kinematics in scapholunate instability: a dynamic CT study

**DOI:** 10.1007/s00256-023-04323-6

**Published:** 2023-03-23

**Authors:** Melanie Amarasooriya, Rami Al-Dirini, Kimberley Bryant, Gregory Ian Bain

**Affiliations:** 1 College of Medicine and Public Health, Flinders University of South Australia, Adelaide, South Australia Australia; 2 College of Science and Engineering, Flinders University of South Australia, Adelaide, South Australia Australia; 3 College of Medicine and Public Health, Flinders University of South Australia, Adelaide, South Australia Australia; 4 Department of Orthopaedic and Trauma Surgery, Flinders University of South Australia and Flinders Medical Centre, Flinders Drive, Bedford Park, Adelaide, South Australia 5042 Australia

**Keywords:** Scapholunate, Dynamic CT, Carpal instability, Scaphoid kinematics

## Abstract

**Objective:**

The scaphoid is proposed to be driven by the distal carpal row in scapholunate instability (SLI) as it is dissociated from the proximal row. The aim of this study was to describe the 6 degrees of freedom kinematics of the scaphoid using dynamic CT in the normal and SLI wrists. We hypothesised that the SLI scaphoid would demonstrate kinematic evidence conforming to distal row motion.

**Materials and methods:**

We studied dynamic CT scans of 17 SLI and 17 normal wrists during ulnar to radial deviation and extension to flexion. The radio-scaphoid angles in three anatomic planes were calculated in the wrist neutral position and during wrist motion. The centroid position was also calculated in the wrist neutral position and during wrist motion. The scapho-capitate motion index (SCI) was calculated as a ratio between the scaphoid and the capitate motion.

**Results:**

In the neutral position of the wrist, the SLI scaphoid was flexed, internally rotated, and radially translated compared to the normal scaphoid. During wrist motion, the SLI scaphoid had more ‘in-plane’ motion and less ‘out-of-plane’ motion with a higher SCI during wrist neutral to radial deviation and extension to neutral.

**Conclusion:**

We have described the malalignment of the SLI scaphoid in the neutral position of the wrist and 6 degrees of freedom kinematics during wrist motion of the SLI scaphoid compared to the normal. The SLI scaphoid conformed more to the distal row motion than the normal scaphoid. This information may help define the surgical reconstruction techniques for SLI.

## Introduction

Scapholunate instability (SLI) is a dissociative carpal instability that occurs when the dorsal scapholunate (dSLL) complex is compromised [1, 2]. Plain radiographic features of SLI include scapholunate diastasis, scaphoid flexion [1], proximal scaphoid dorsal subluxation [3], and dorsal intercalated segmental instability (DISI) [1]. The 3-dimensional computed tomography (3D-CT) provides a better perspective of the findings and allows an appreciation of the internal rotation of the scaphoid [4, 5]. 3D-CT also enables the 3D quantification of carpal malalignment in static positions of the wrist.

Malalignment and instability, however, are distinctly different concepts [5]. The wrist joint is unstable if it is symptomatic, is not able to bear loads, and does not exhibit normal kinematics during any portion of its arc of motion [6]. Dynamic studies including stress views and dynamic fluoroscopy can accentuate or unmask the instability [7].

Initial research into dynamic changes in SLI was conducted using uniplanar and later biplanar radiography [8, 9]. Electromagnetic, optical, and radio-opaque markers have been used to track carpal bones with wrist motion [10–12]. Dynamic tracking of carpal bone motion on cadavers has defined the carpal instability patterns following serial sectioning of carpal ligaments [13]. Surgically implanted markers/sensors, limit these studies to be conducted on cadavers. Surgically created ligament injuries in cadavers will respond differently to the traumatic injuries observed in our patients’ wrists.

3D imaging studies combined with marker-less registration techniques have facilitated in vivo assessment of the carpal instabilities [14]. 3D-CT-based studies only allow a limited number of static positions to be studied and kinematics in between extrapolated [15, 16]. Biplanar videoradiography (BVR) and dynamic CT (4D-CT) both overcome these limitations by combining the strengths of 3D-CT with marker-less registration and representing true dynamic motion in vivo [17, 18]. BVR has been proven to be accurate to define in-vivo carpal kinematics. However, an inherent limitation is difficulty in the tracking of carpal bones when they overlap [17]. Other concerns with BVR are its limited availability and the time-consuming specialist post-processing assessment.

4D-CT is now clinically more available and a validated method to track carpal bones with sub-millimetre and sub-degree precision and accuracy [19]. The radiation dose for a 4D-CT scan is 0.231 mSv [18], which is considered low-dose radiation. Recent 4D-CT publications have been directed at improving the diagnostic accuracy of dynamic SL instability [20–22]. However, there have been no reports on the 6 degrees of freedom kinematics of the scaphoid in SLI in an in vivo study.

The aim of this study was to assess and compare the in vivo scaphoid 6 degrees of freedom kinematics in normal and SLI wrists using 4D-CT. This includes:

- The scaphoid alignment in the wrist neutral position
- Kinematic assessment of the scaphoid during extension to flexion, and ulnar to radial deviation.

We expected that dynamic CT will provide a better understanding of the scaphoid kinematics in the normal and SLI wrist. Once dissociated from the lunate, the scaphoid is theorised to be driven by the distal carpal row [23]. We hypothesised that the SLI scaphoid would demonstrate kinematic evidence conforming to the capitate motion compared to the normal scaphoid. The findings of this study may help in further defining the surgical reconstruction techniques for SLI.

## Methods

### Participants

After institutional review board approval, dynamic CT scans of patients with SLI were selected from the database maintained at the Flinders Medical Centre Upper Limb Unit. Inclusion criteria were any non-arthritic stage of SLI, confirmed by wrist arthroscopy performed by fellowship qualified upper limb surgeon. Patients’ dynamic scans were excluded if aged under 18 years, pregnant, or there were radiographic or arthroscopic evidence of degenerative changes and evidence of complex wrist injuries (e.g. associated fractures, peri lunate dislocations). Seventeen patients fulfilled the above criteria (*n* = 17).

Seventeen consecutive normal right wrist scans (*n* = 17) were identified from an anonymized database maintained at the Monash Health Radiology Department. These scans were performed on healthy volunteer participants between the ages of 18–30 years with no pre-existing wrist pathology. The data was archived and anonymized with no demographic details to comply with the ethics agreement; therefore, case matching was not performed as demographic data was unavailable for the normal samples.

### CT scanning

CT scanning for both normal and SLI wrists followed a previously published protocol [18]. The wrist was placed in the CT scanner gantry. A scout image was performed with the wrist in the neutral position to centre the carpus in the gantry. The patients were then, instructed to move the wrist from ulnar to radial deviation and back to the neutral position, followed by extension to flexion. Images were acquired with an axial slice thickness of 0.6 mm, at 4 frames per second. The output from the scanner was archived as raw data in DICOM (Digital Imaging and Communication in Medicine) format. The effective radiation dose was calculated as 0.231 mS^9^.

### Data management

**Fig. 1:**
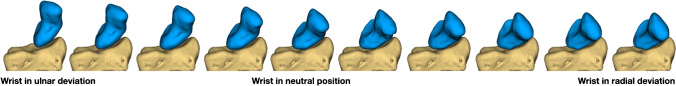
Scaphoid positions during wrist ulnar to radial deviation, with its relation to the radius. The scaphoid is segmented at each time point and saved as a 3-dimensional mesh in the stereolithography (STL) format. The image shows the relationship of the scaphoid to the radius. Every 4th consecutive frame/time point of a single representative normal wrist is depicted

Raw DICOM data were transferred to open-source software, 3D slicer (http://www.slicer.org) [24] for analysis. DICOM data were converted to discrete 3D volumes for each acquisition time point. Each 3D volume (frame) was separately segmented to generate surface-rendered meshes (stereolithography-STL format) for each carpal bone (Fig. 1). The segmentation followed the steps described by Zhao et al. [19] and time taken for segmentation of each is estimated between 8 and 10 h.

### Geographical representation

**Fig. 2:**
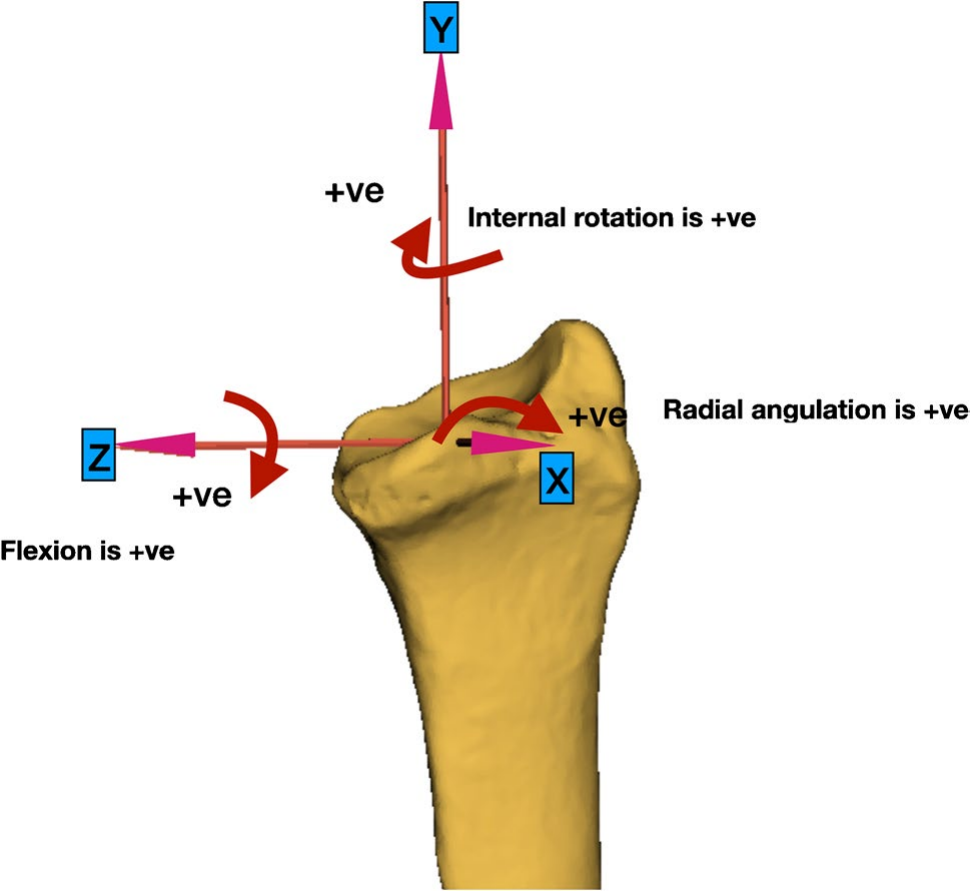
Radius coordinate system and definitions of directions of motion. The iso-centre was at the midpoint of the ridge between the scaphoid and the lunate facets. The *y*-axis is the longitudinal axis of the radius. The *z*-axis is the line perpendicular to the y-axis, passing from the iso-centre to the radial styloid. The *x*-axis is orthogonal to *z* and *y*. Positive rotation is illustrated with arrows in flexion, radial, and distal directions

The radius was defined as the reference bone. The radius coordinate system was placed following the International Society of Biomechanics (ISB) guidelines [25, 26] (Fig. 2). The directions were modified to indicate ulnar positive along the *z*-axis, distal positive along the *y*-axis and volar positive along the *x*-axis. Extension to flexion (flexion + ve) was defined as the rotation around the *z*-axis [27]. The ulnar to radial deviation (radial + ve) was defined as the rotation around the *x*-axis**.** Internal to external rotation (internal rotation + ve) was defined as the rotation around the *y*-axis. When the left wrist was analysed, it was mirrored to reflect the right wrist, so that the defined axes and directions remained consistent. The neutral position of the wrist was defined by the position where the longitudinal axis of the 3rd metacarpal aligns with the longitudinal axis of the radius.

### Linear and angular displacement

The individual carpal bone models were registered using a rigid iterative closest point algorithm on the 3D slicer platform [24]. The fixed model of the carpal bone was the bone at the neutral position of the wrist. The moving model was the carpal bone model in a subsequent wrist position. The rigid registration of the scaphoid from the moving model to the fixed model generates a linear transformation matrix [28]. The resultant linear transformation matrix was used to calculate the displacement field between the individual carpal bone positions pertaining to each time point [29]. The calculated scaphoid angular displacement was expressed as Euler angles using an intrinsic rotation system with the sequence of rotation around the *z*-axis, followed by the *x*-axis, followed by the *y*-axis [25].

### Angular displacements

The radiocarpal angles in each plane were defined by the angle between the primary principal axis of the carpal bone and the *x*-, *y*-, or *z*-axis of the radius coordinate system as described by Coburn et al. [30]. For the moving wrist, the full length of the 3rd metacarpal was not always visible. As the capitate posture closely resembles the 3rd metacarpal posture, the radio-capitate angle was used as a surrogate for the wrist angle [31] for the moving wrist.

### Linear displacements

The position of the centroid of the scaphoid was used to describe the linear displacement as described by previous researchers [4, 11].

### Motion index relative to capitate

Capitate was considered representative of the distal carpal row. Therefore, scaphoid motion relative to the capitate (scapho-capitate motion index-SCI) was calculated [32] to quantify the conformity of the scaphoid motion to the capitate motion.

$$Scapho- capitate\ motion\ index\ (SCI)=\left(\frac{Scaphoid\ motion}{Capitate\ motion}\right)\times 100\%$$

It is calculated based on the “in-plane” scaphoid motion and presented as a percentage. An SCI of 100% would indicate that the scaphoid and capitate motion are the same in magnitude in the same plane. Hence, the scaphoid is fully conforming to capitate (distal row) motion in the studied plane.

### Statistical analysis

Measurements and calculations were generated using previously described software techniques [19, 33] without manual measurements or observations. The largest angular error for scaphoid registration was reported to be around *y*-axis and the mean was 0.4° (± 0.9°) [19]. The largest translational error was reported to be along *z*-axis and the mean was 0.038 mm (± 0.22). We performed test and retest repeatability to assess the precision of the registration and the ICC (interclass correlation coefficient) was found to be excellent (0.99). Linear interpolation of the data was performed using custom scripts (Matlab version Rb2020®, Mathworks, USA), which created a comparable set of data points for statistical analysis. Graphs were created on Microsoft Excel® 2016. The standard statistical parameters were calculated using STATA Macintosh version 17.0. A Shapiro-Wilk’s test was used to ascertain normal distribution of the results followed by an unpaired *t*-test to compare the means and standard deviations. The statistical significance was determined at a *p*-value less than 0.05.

## Results

Dynamic CT scans of the 17 normal wrists were all right wrists from participants between the age of 18–30 years. Their gender is unknown. Seventeen SLI patients included 14 males with a mean age of 33.6 years (± 13) and three females with a mean age of 38 ± 7 years. There were 10 right and 7 left wrists. No patients had degenerative changes on imaging or arthroscopy.

### Wrist range of motion

The mean range of motion was less for the SLI wrist than the normal wrist, in both extension to flexion and in ulnar to radial deviation (Table 1). This was statistically significant with extension to flexion, (mean ± standard deviation, SLI 85.9° ± 29.7° vs normal 119.6° ± 24.3° *p* < 0.01).

**Table 1:** Wrist ranges of motion

Wrist motion	Normal (*n* = 17)		SLI (*n* = 17)		*p*-value
	Mean ± SD	95% CI	Mean ± SD	95% CI	
Wrist extension to flexion	119.6° ± 24.3°	107–132°	85.9° ± 29.7°*	70.6–101.2°	< 0.01
Extension (extension to neutral)	65.9° ± 12.6°	59.4–72°.4	52.2° ± 13.9°*	45–59.4°	< 0.01
Flexion (neutral to flexion)	53.7° ± 20.7	43–64.3°	33.7° ± 18.9°*	24–43.4°	< 0.01
Wrist ulnar to radial deviation	53.3° ± 7.2°	49.6–57°	47.3° ± 10.9°	41.7–53°	0.07
Ulnar deviation to neutral	32.2° ± 7.8°	28.2–36.2°	27.6° ± 9.3°	22.9–32.4°	0.13
Neutral to radial deviation	21.1° ± 7.2°	15.3–24°	19.7° ± 8.5°	15.3–24°	0.61

Note that the mean range of motion was less for the SLI wrist for all measures. A statistically significant difference was seen during wrist extension to flexion. ‘*’ Indicate statistical significance at a *p*-value of < 0.05

### Scaphoid angulations

#### Wrist in neutral position

**Fig. 3:**
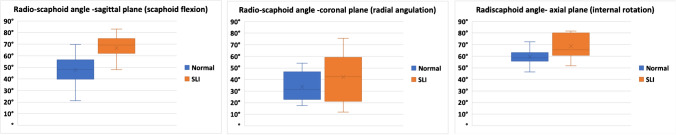
The radio-scaphoid angle of the normal and the SLI wrists with the wrist in the neutral position, in three anatomic planes, coronal, sagittal, and axial. Note that the SLI scaphoid is more flexed and internally rotated than the normal scaphoid

The SLI scaphoid was more flexed (66.5° ± 13.1° vs 47.8° ± 13.2°, *p*<0.01) and internally rotated (68.8° ± 10.6° Vs 59.4° ± 7.7°, *p* < 0.01) than the normal scaphoid (Table 2 and Fig. 3). There was no significant difference in the radial angulation between the two groups.

**Table 2:** The radio-scaphoid angles with the wrist in the neutral position

Rotation	Normal		SLI		*p*-value
	Mean ± SD	95% CI	Mean ± SD	95% CI	
Radial angulation (coronal plane)	33.8° ± 12.5°	27.3–40.1°	42.3°± 21.1°	31.4–53.1°	0.16
Flexion (sagittal plane)	47.8° ± 13.2°	41–54.6°	66.5°± 13.1°*	59.7–73.2°	< 0.01
Internal rotation (axial plane)	59.4° ± 7.7°	55.4–63.4°	68.8°± 10.6°*	63.3–74.2°	< 0.01

The results presented are the mean and ±standard deviation. ‘*’ Indicate statistical significance at a *p*-value of < 0.05

#### Wrist ulnar to radial deviation

**Fig. 4:**
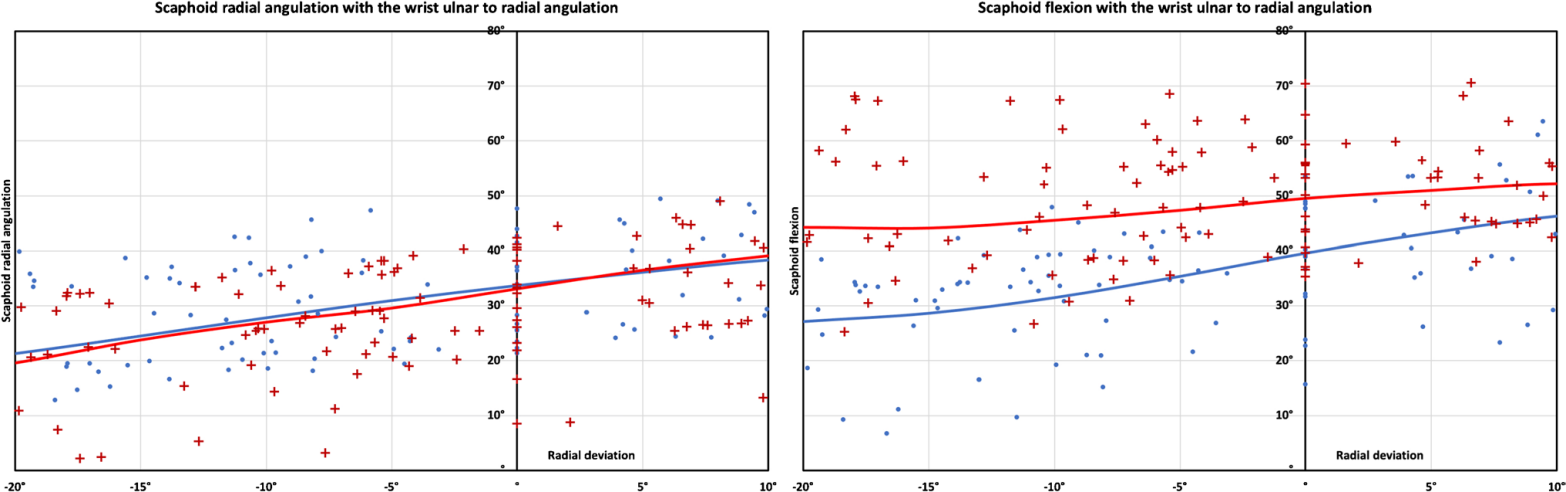
Scaphoid angulations during the wrist ulnar to radial deviation a. Scaphoid radial angulation. (in-plane motion) The SLI scaphoid had more radial angulation (in-plane motion) (*p* < 0.05) than the normal scaphoid. b. Scaphoid flexion (out-of-plane motion). The SLI scaphoid is more flexed than the normal scaphoid. The flexion arc of the SLI scaphoid is less than the flexion arc of the normal scaphoid (*p* < 0.01). The results presented are the radio-scaphoid angle, with the wrist moving from radial to ulnar deviation. The markers indicate the individual raw data points, and the lines are the mean radio-scaphoid angle following linear interpolation

**Fig. 5:**
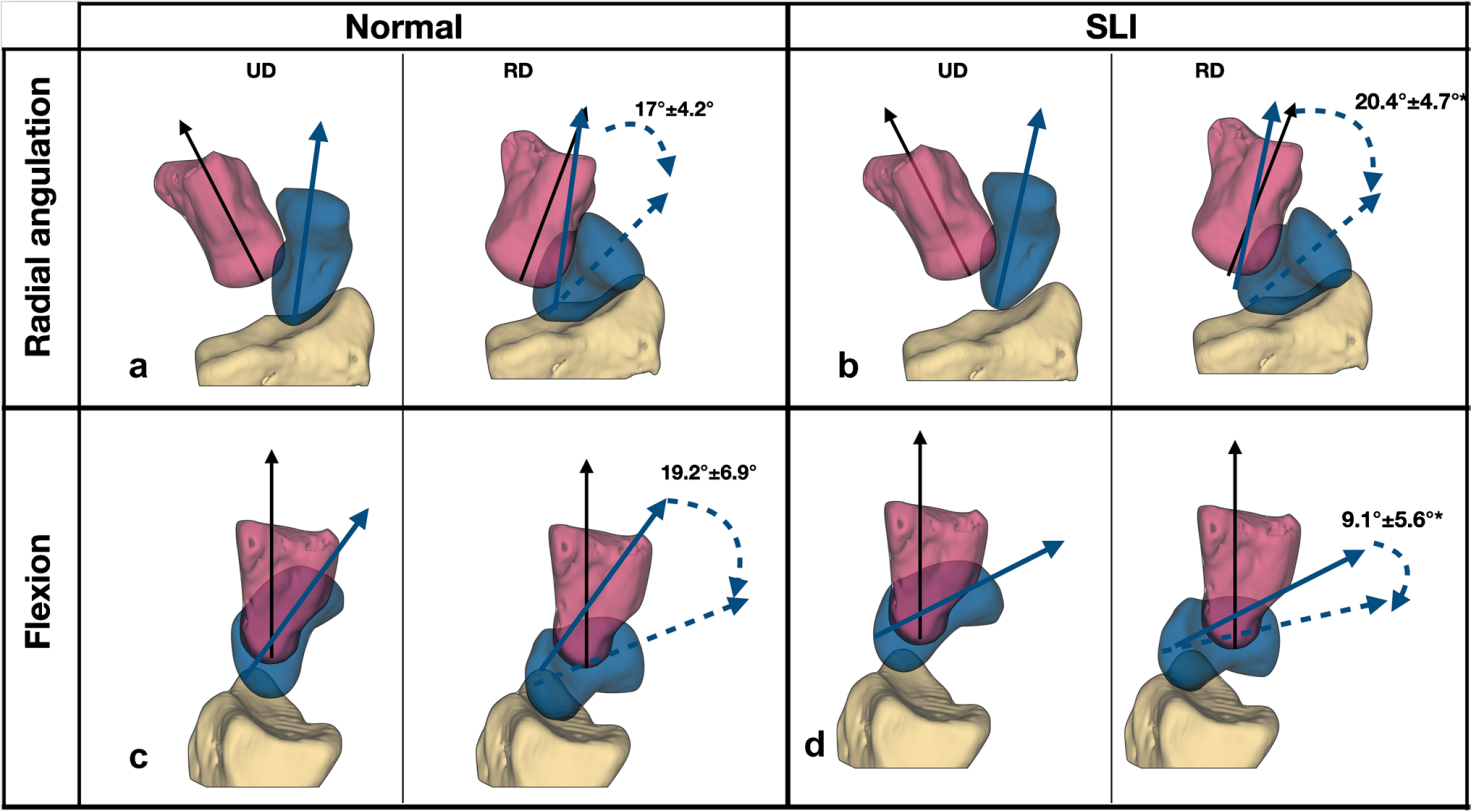
Scaphoid angulations during wrist ulnar to radial deviation. Scaphoid radial angulation (in-plane motion), **a** normal 17° ± 4.2° (SCI 56.8%). **b** SLI 20.4° ±4.7°, (SCI 68%). *(*p* < 0.05). Flexion (out of plane motion). **c** Normal 19.2° ±6.9° d. SLI 9.1° ±5.6°* (*p* < 0.01). The wrist is moving from 20° ulnar to 10° radial deviation, indicated by the black arrow on the capitate. Images are from a single representative right wrist (volar **a** and **b**, ulnar **c** and **d** views) with mean and standard deviation for the arc of scaphoid motion mentioned

With the wrist ulnar to radial deviation, the SLI scaphoid had less flexion (out of plane motion) than the normal scaphoid (SLI 9.1° ± 5.6° Vs normal 19.2° ± 6.9°, *p* < 0.01; Fig. 4 and Fig. 5). The SLI scaphoid had more radial angulation than the normal scaphoid (SLI 20.4° ± 4.7° Vs 17° ± 4.2°, *p* < 0.05). Internal rotation was not significantly different between the normal and the SLI groups.

With the ulnar to radial deviation of the wrist, the scaphoid radial angulation is the “in-plane motion” of the scaphoid. The SCI for the SLI wrist was significantly higher than the normal wrist (SLI 68% ± 15.7 Vs normal 56.8% ± 13.9, *p* < 0.05; Table 3). The SCI was significantly higher in SLI (*p* < 0.01) during neutral to radial deviation but not during ulnar deviation to neutral.

**Table 3:** Scapho capitate motion index (SCI) during wrist ulnar to radial deviation

Rotation	Normal	SLI	*p*-value
Ulnar to radial deviation	56.8% ± 13.9	68% ± 15.7*	< 0.05
Neutral to radial deviation	46.5% ± 15	65.9% ± 16.1 *	< 0.01
Ulnar deviation to neutral	62% ± 17.7	69.1% ± 19.6	0.28

The results presented are the mean and ± standard deviation. ‘*’ Indicate statistical significance at a *p*-value of < 0.05

#### Wrist extension to flexion

**Fig. 6:**
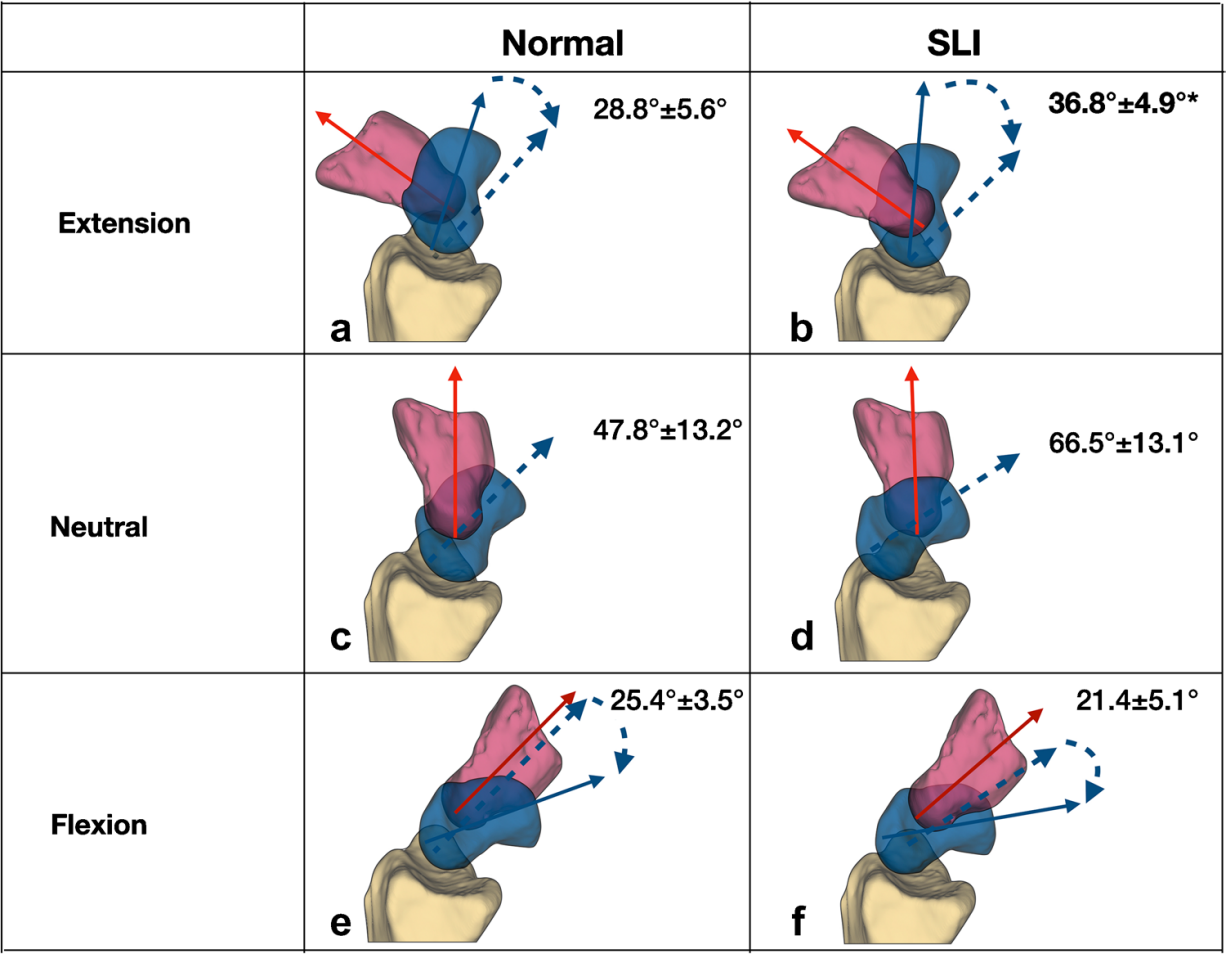
Scaphoid angulations during wrist extension to flexion. During wrist extension to neutral, there was more scaphoid flexion in the SLI wrists. SLI wrists had a greater scapho-capitate motion index than the normal wrist. **a** Normal 28.8° ± 5.6°, 92%, **b** SLI 36.8° ± 4.9, 71%. *p* < 0.01. The wrist in the neutral position, the SLI scaphoid (**d**) was more flexed than the normal scaphoid (**c**) (SLI 66.5°± 13.1° vs normal 47.8° ± 13.2° *p* < 0.01) During wrist flexion, the SLI wrist motion was not significantly different. **e** Normal 25.4° ± 3.5°, 82%, **f** SLI 21.4° ± 5.1°, 71%, *p* = 0.16. The results presented are the arc scaphoid motion, indicated by the curved blue arrow. Images are the ulnar views of a single representative right wrist with mean and standard deviation

**Fig. 7:**
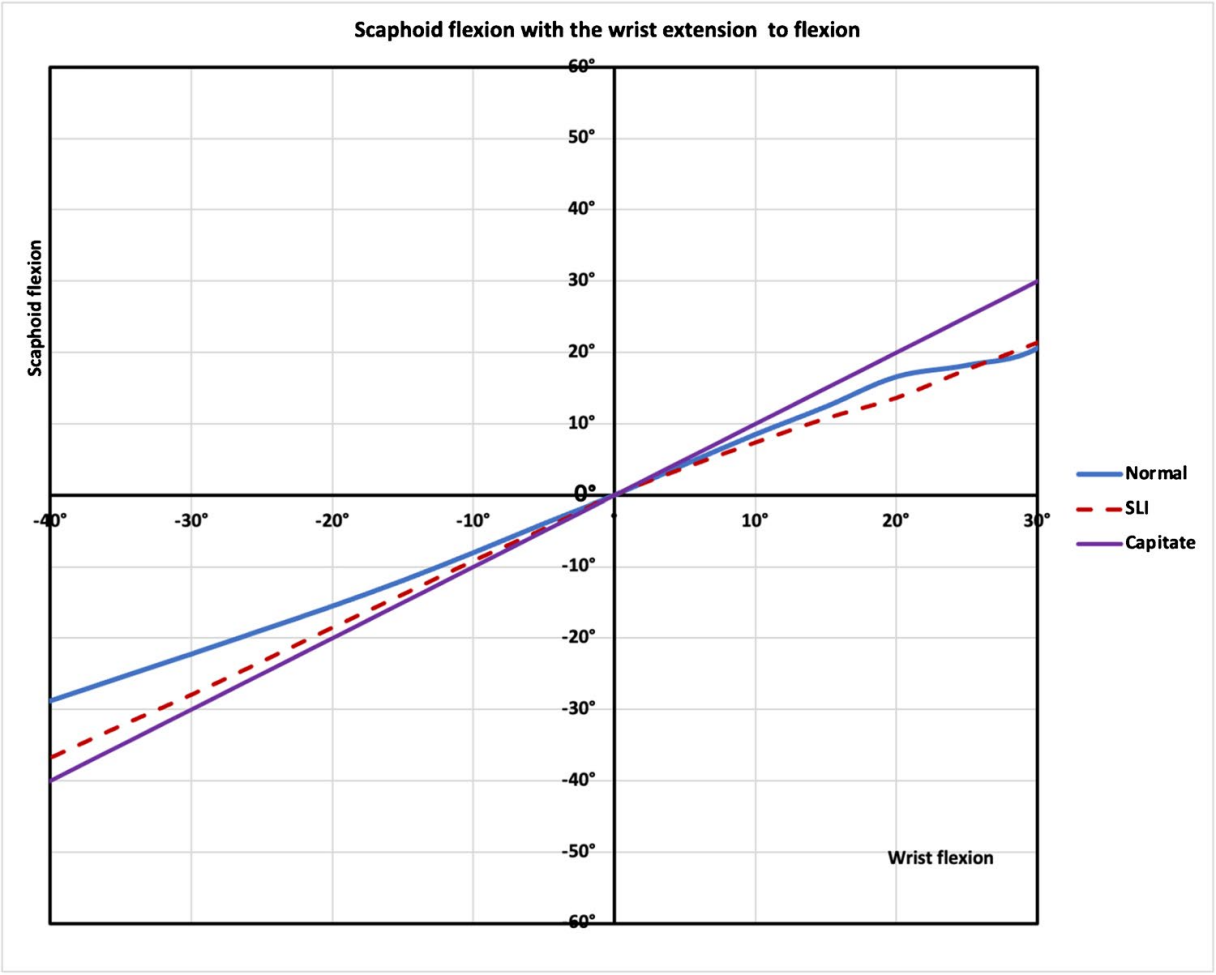
Scaphoid flexion during wrist extension to flexion. During wrist extension to the neutral position, the ‘in-plane motion of the SLI scaphoid’ (SLI-red) almost resembles the capitate motion (capitate-purple). so that the scaphoid flexion closely follows the capitate motion. The SLI scaphoid has a higher scapho-capitate index (SCI) during wrist extension to the neutral position (*p* < 0.01)

During the wrist 40° extension to the neutral position, the SLI scaphoid had more flexion arc than the normal scaphoid (SLI 36.8° ± 4.9° vs normal28.8° ± 5.6°, *p* < 0.01; Fig. 6). The SCI for SLI was significantly higher than for the normal wrist during extension to neutral (SLI 92.0% ± 12.2, normal 72 % ± 13.9, *p* < 0.01; Fig. 7). From wrist extension to flexion, radial angulation of the scaphoid or the internal rotation was not significantly different between the normal and SLI wrists**.**

### Scaphoid centroid position

#### Wrist in neutral position

The scaphoid centroid is more radial in SLI than in the normal wrist (*p <0.05*; Table 4). There were no statistically significant differences in the proximodistal or dorso-volar planes.

**Table 4:** The scaphoid centroid position in the wrist neutral position

Direction	Normal		SLI		*p*-value
	Mean ± SD	95% CI	Mean ± SD	95% CI	
Radio-ulnar (along the ***z***-axis ulnar + ve)	− 6.2± 1.2	5.6–6.9	− 7.2 ± 1.3*	6.5–7.8	< 0.05
Dorso-volar (along the ***x***-axis volar + ve)	3.7 ± 1.3	3–4.3	3.6 ± 0.8	43.2–4	0.84
Proximo-distal (along the ***y***-axis distal + ve)	13.4 ± 1.6	12.5–14.2	13.9 ± 1.9	12.8–14.8	0.42

The results presented are the mean and ± standard deviation. ‘*’ Indicate statistical significance at a *p*-value of < 0.05. Ulnar is + ve; hence, negative values indicate radial direction. The measurements are in mm

#### Wrist ulnar to radial deviation

The scaphoid centroid translations were not different between the normal wrist and the SLI wrist in any plane during the wrist ulnar to radial deviation (Table 5).

**Table 5:** Scaphoid centroid translations during the wrist ulnar to radial deviation

Translation	Normal	Normal	*p*-value
Radioulnar (along the ***z***-axis ulnar + ve)	1.1 ± 0.7	1.1 ± 0.6	0.94
Dorso-volar (along the ***x***-axis volar + ve)	1.1 ± 0.6	0.7 ± 0.8	0.15
Proximo distal (along the ***y***-axis distal + ve)	− 3.1 ± 0.9	− 3.5 ± 0.8	0.23

The results presented are the mean and ±standard deviation

#### Wrist extension to flexion

With wrist extension to flexion, the SLI scaphoid centroid proximally translated more than the normal scaphoid (SLI 3.2 mm ± 0.6 mm vs normal 1.9 mm ± 1.1 mm, *p* < 0.01; Table 6). There were no statistically significant differences in the other planes.

**Table 6:** Scaphoid centroid translations during the wrist extension to flexion

Translation	Normal	Normal	*p*-value
Radio-ulnar (along the ***z***-axis ulnar + ve)	0.3 ± 0.6	0.7 ± 0.8	0.17
Dorso-volar (along the ***x***-axis volar + ve)	2.7 ± 1.1	3.0 ±1.5	0.61
Proximo-distal (along the ***y***-axis distal + ve)	− 1.9 ± 1.1	− 3.2 ± 0.6*	< 0.01

The results presented are the mean and ±standard deviation. ‘*’ Indicate statistical significance at a *p*-value of < 0.05. Distal is + ve; hence, negative values indicate proximal direction. The measurements are in mm

## Discussion

The aim of the current study was to assess and compare the in-vivo scaphoid kinematics in the normal and SLI wrists and to describe them in six degrees of freedom. We hypothesised that the SLI scaphoid would demonstrate objective evidence of conforming to the capitate rotation compared to the normal scaphoid.

Our study confirmed that in the neutral wrist position, the SLI scaphoid is more flexed, internally rotated, and radially translated. Our results are similar to previous studies on the malalignment in the SLI scaphoid [4, 13, 34, 35]. Werner et al. reported that in a cadaveric model, the scaphoid internally rotated less than 4° following the sectioning of SLIL and volar or dorsal ligaments. Omori et al. in an in vivo study of 3 cases using 3D-CT reported that the SLI scaphoid is internally rotated by 7° compared to the normal [4]. Our findings were similar in the direction of motion, and more in accordance with Omori et al. on the magnitude of rotation.

**Fig. 8:**
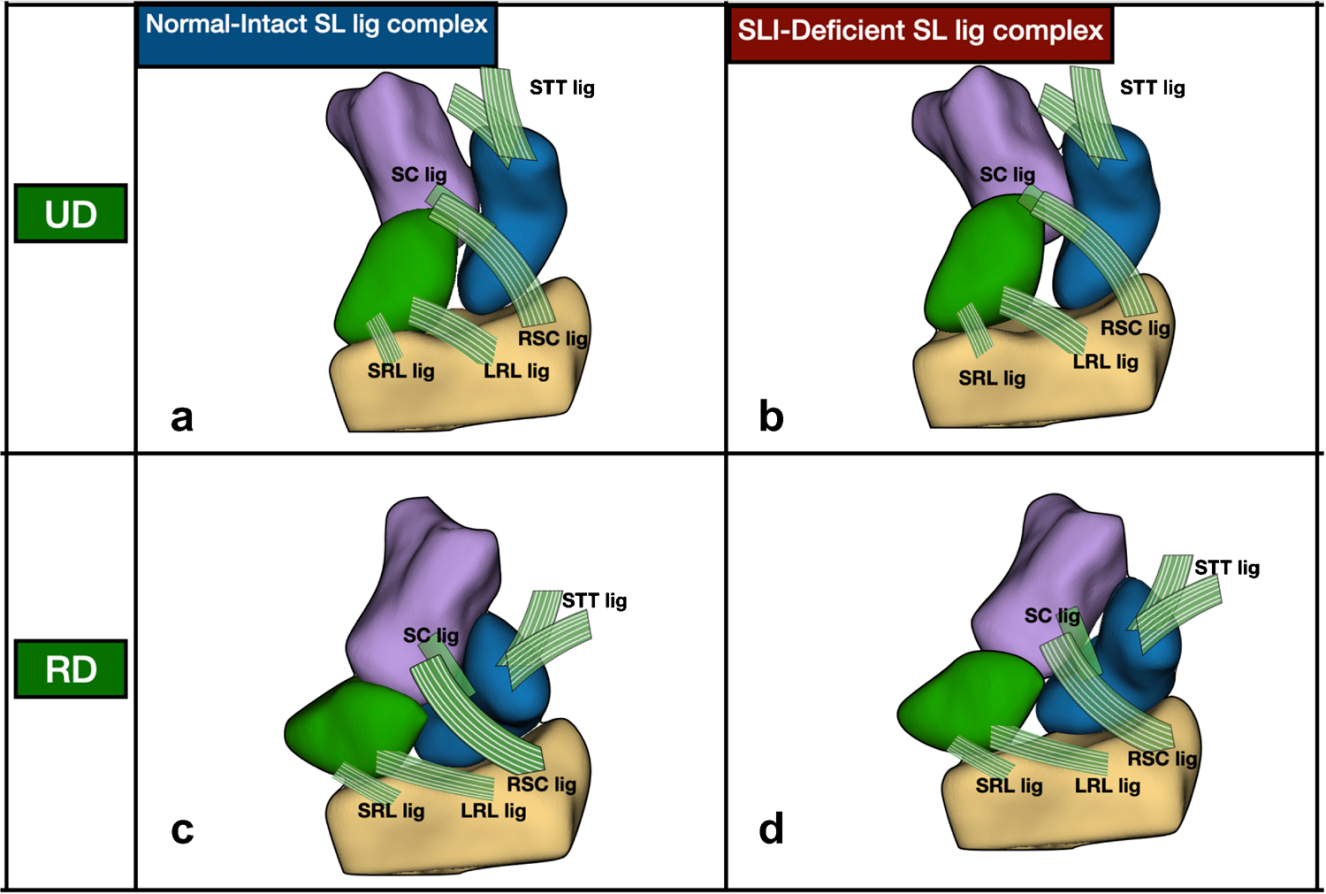
Possible explanation for kinematic changes seen in the SLI. During wrist ulnar to radial deviation the normal scaphoid (**a** and **c**) naturally flexes in an “out of plane” motion. This is likely due to the influence of the SL ligament and DIC. With SLI, the proximal pole becomes unstable, as the SLL and DIC have been disrupted. As the distal restraints are likely to be intact, they provide a deforming force for the scaphoid, leading to less out-of-plane motion and more in-plane motion (**b** and **d**). This is reflected in the increased scaphoid capitate angulation index. Images are the volar views of a single normal wrist and a single SLI wrist

During wrist motion, we found that the SLI scaphoid was more associated with capitate rotation than in normal wrists. With radial deviation of the wrist, the flexion arc of the SLI scaphoid was less, and the radial deviation arc was more indicating more in-plane motion and less out-of-plane motion. A possible explanation is that the radial-sided wrist and finger tendons act with the trapezium and trapezoid on the distal pole of the scaphoid, forcing the lateral column to shorten. In SLI the proximal pole is ‘unrestrained’ and not linked to the lunate (Fig. 8). The scaphoid therefore follows the capitate and tends to move in-plane with the distal row during radial deviation.

In the normal wrist, as the radial column shortens, the proximal pole is restrained by the SL complex. The scaphoid hinges on the dSLL, moving into flexion, in an out of plane motion. Thus, the distal moments acting on the distal pole are balanced by the dorsal SL complex, directing the normal scaphoid into obligatory flexion. In the normal wrist, with the DIC and dSLL intact, the proximal scaphoid motion will be associated with the lunate and restrained by the lunate.

In a recent cadaveric study, Figueroa et al. reported that the flexion arc for the SLI scaphoid is less than the normal scaphoid during wrist radioulnar deviation [36]. Our findings agree with Figueroa et al.; we have also described the scaphoid rotation in other planes (radioulnar and internal rotation) between the normal and SLI patients.

During wrist extension to the neutral position, the SLI scaphoid was more associated with the capitate extension with an SCI of 92% compared to the normal scaphoid which had an SCI of 72%. Our findings on the normal scaphoid are similar to the SCI of 74% reported by Rainbow et al in a 3D-CT study [32]. However, there was no published literature on SCI on SLI wrists. A cadaveric study by Figueroa et al. did not find a significant difference in the scaphoid flexion between the normal and SL deficient wrists during wrist extension to flexion. However, they have not separated the flexion and extension phases of the wrist [36].

A strength of our study was that we assessed the extension to neutral and neutral to flexion phases separately and compared them between the normal and SLI wrists. We identified that the SLI scaphoid conforms more to the capitate motion in the extension phase but not in the flexion phase. It is possible that the flexor carpi radialis (FCR) acts as a stabilising force in the flexion [37] so that the SLI scaphoid does not conform to the capitate rotation fully during flexion.

To enable an effective comparison between the wrists, we assessed the range from 20° ulnar deviation to 10° radial deviation and 40° extension to 30° flexion. This is a limitation of the study and could be a potential reason for not identifying a significant difference in the change of internal rotation of the scaphoid during wrist motion. In addition, the patients belong to a heterogenous group including all stages of non-arthritic SLI. With the limited number of 17 cases, we are not able to comment on the differences between each stage. While the limitations may affect the applicability of the findings to every single case, our findings can be used to identify which cases fit into the pattern described in the current study.

In conclusion, the current study has demonstrated features of malalignment in the neutral position and the kinematic changes during motion in the SLI scaphoid in 6 degrees of freedom. The scaphoid moves more in-plane with the capitate during the wrist extension and radial deviation. The SLI scaphoid behaves more as a distal row bone conforming to the in-plane wrist motion, which was measured by the scaphoid capitate motion index. With the findings of the study, we believe that most of the patients may benefit from reinforcing the dSLL complex alone, without needing to reinforce the ligamental restraints between the scaphoid and the distal row. However, clinical follow-up of surgical techniques is necessary before any recommendations on surgical techniques are made.
